# Agricultural Soil as a Reservoir of *Pseudomonas aeruginosa* with Potential Risk to Public Health

**DOI:** 10.3390/microorganisms12112181

**Published:** 2024-10-30

**Authors:** Jessica I. Licea-Herrera, Abraham Guerrero, Maribel Mireles-Martínez, Yuridia Rodríguez-González, Guadalupe Aguilera-Arreola, Araceli Contreras-Rodríguez, Susana Fernandez-Davila, Rocío Requena-Castro, Gildardo Rivera, Virgilio Bocanegra-García, Ana Verónica Martínez-Vázquez

**Affiliations:** 1 Centro de Biotecnología Genómica, Instituto Politécnico Nacional, Reynosa 88710, Tamaulipas, Mexico; jliceah1800@alumno.ipn.mx (J.I.L.-H.); mmireles@ipn.mx (M.M.-M.); yrodriguezg2300@alumno.ipn.mx (Y.R.-G.); sfernandezd@ipn.mx (S.F.-D.); rrequenac_23@hotmail.com (R.R.-C.); giriveras@ipn.mx (G.R.); vbocanegra@ipn.mx (V.B.-G.); 2 Consejo Nacional de Ciencia y Tecnología (CONAHCyT), Centro de Investigación en Alimentación y Desarrollo (CIAD), Mazatlán 82100, Sinaloa, Mexico; aguerrero@ciad.mx; 3 Escuela Nacional de Ciencias Biológicas, Instituto Politécnico Nacional, México City 11340, Mexico; lupita_aguilera@hotmail.com (G.A.-A.); acontrerasr@ipn.mx (A.C.-R.)

**Keywords:** *Pseudomonas*, agriculture, virulence, antimicrobial resistance, México

## Abstract

*Pseudomonas aeruginosa* is an opportunistic pathogen with a high capacity to adapt to different factors. The aim of this study is to analyze the pathogenicity in *P. aeruginosa* strains and their resistance to heavy metals and antibiotics, in agricultural soil of the state of Tamaulipas, Mexico. Susceptibility to 16 antibiotics was tested using the Kirby-Bauer method (CLSI). Eight virulence factors (FV) and six genes associated with heavy metal resistance were detected by PCR. As a result, *P. aeruginosa* was detected in 55% of the samples. The eight virulence factors were identified in ≥80% of the strains. The strains showed some level of resistance to only three antibiotics: 32.8% to ticarcillin, 40.8% to ticarcillin/clavulanic acid and 2.4% to aztreonam. The most frequent heavy metal resistance genes were *ars*C (92.8%) and *cop*A (90.4%). However, *cop*B and *ars*B genes were also identified in a percentage greater than 80%, and the least frequent genes were *mer*A in 14.4% and *czc*A in 7.2%. Although *P. aeruginosa* strains showed a high percentage of factor virulence (potential ability to cause infections), their high levels of susceptibility to antibiotics lead to the assumption that infections are easily curable.

## 1. Introduction

*Pseudomonas aeruginosa* is the most prevalent opportunistic pathogen of environmental origin and is a major cause of bloodstream infections, ventilator-associated pneumonia, nosocomial urinary tract and surgical site infections [1,2,3]. It is the cause of 18–61% of hospital deaths due to nosocomial infections [4,5]. *Pseudomonas* spp. infections are difficult to treat owing to multiple factors, including the ability to cause direct damage to host tissue, high genomic plasticity, extensive intrinsic drug resistance and the progressive increase in antimicrobial resistance [6]. Thus, this bacterium has multiple antibiotic resistance phenotypes that could allow its survival during antibiotic treatment of an infection [7]. Therefore, *P. aeruginosa* has been included as one of the three bacterial species on the list of “priority pathogens” that urgently require the development of new treatment alternatives by the World Health Organization [8,9]. Its pathogenic profile stems from the large and variable arsenal of virulence factors and antibiotic resistance determinants harbored in *P. aeruginosa’s* genome, which confer remarkable ability to adapt to multiple conditions [1,10,11,12,13]. Although most studies on *P. aeruginosa* have been conducted in a hospital environment (clinical samples, utensils, surfaces, equipment and others), it is considered that this is only a temporary habitat, and that contamination has another origin [14]. That is why several studies have focused on studying soil, water, plants and animals as natural and permanent reservoirs for the bacteria [14,15,16].

Furthermore, as has been discussed in several studies, human, industrial and agricultural activities are intensifying the environmental resistome (i.e., the total collection of all genes that can directly or indirectly confer resistance to antibiotics in the environment) [15,17,18]. In this way, the increase in antibiotic resistance genes in the environment is stimulated, which further increases the number of resistant pathogenic microorganisms in different environments, thus endangering human health [19]. The mechanism of emergence and spread of antimicrobial resistance genes is a complex process, which includes multiple factors such as heavy metals [20], the aggressive use of antimicrobials in the intensive agriculture and livestock farming [21], wastewater treatment plant (WWTP) disposal [22] and use of antibiotics either as growth promoters and/or disease prevention of animals [23,24].

The proportion of *P. aeruginosa* strains demonstrating multidrug resistance is increasing worryingly worldwide [25]. In different countries, the prevalence of multidrug-resistant isolates varies from 14% to 30%, with carbapenem resistance occurring in 15–22% of cases, these isolates being those included in the WHO priority lists [6,25,26]. However, this antimicrobial resistance and virulence of *P. aeruginosa* strains may vary between different regions depending on various factors.

Given its ubiquitous distribution, its ability to infect/colonize a variety of hosts and its capacity for resistance to antibiotics, *P. aeruginosa* can be considered a model to study from a “one health” perspective [27,28]. In Mexico, few studies address the presence of *Pseudomonas aeruginosa* in the environment, so little is known about its resistance to antibiotics or heavy metal, and virulence. Therefore, the aim of this study was to evaluate the resistance to heavy metal and antibiotics, as well as the pathogenicity in *P. aeruginosa* strains from agricultural soil in the state of Tamaulipas, Mexico.

## 2. Materials and Methods

### 2.1. Samples Collection

A total of 100 agricultural soil samples were collected at different sites in the state of Tamaulipas, Mexico, in the years 2021–2023. In each site, samples of soil were taken at 20 cm depth. Each sample was placed individually in a plastic bag, labeled and stored in an icebox for transport to a laboratory in Centro de Biotecnología Genómica-IPN. 

### 2.2. Isolation and Identification of Pseudomonas aeruginosa

All the samples were homogenized in peptone water (1:9 proportion) (Becton Dickson & Co., Cuautitlán Izcalli, Mexico) and incubated for 18–24 h at 37 °C. Following homogenization, samples were inoculated in triplicate on CHROMagar *Pseudomonas* (CHROMagar, Paris, France) plates and incubated overnight at 37 °C. After incubation, presumptive colonies with morphological characteristics of *P. aeruginosa* were inoculated individually on trypticase soy agar (TSA) plates (Becton Dickson & Co., Cuautitlán Izcalli, Mexico) and incubated for 24 h at 37 °C. 

All the strains’ identities were confirmed using two methods: (a) Polymerase Chain Reaction (PCR) and (b) MALDI-TOF (Matrix-Assisted Laser Desorption/Ionization Time-of-Flight Mass Spectrometry):

(a) PCR identification: DNA was extracted from bacterial culture using the cell lysis method [29]. One-day-old colonies were picked, suspended in 500 µL MiliQ water and lysed by incubation at 95 °C for 15 min. Afterwards they were centrifugated at 13,000 for 3 min. The supernatant was used to perform the PCR. DNA was stored at −20 °C until use.

The specific amplification primer used for PCR assay were *rpo*D (PsEG30F, 5′-ATYGAAATCGCCAAR CG-3′; PsEG790R, 5′-CGGTTGATKTCCTTGA-3′) for the genus *Pseudomonas* and *ecf*X (ECF5, 5′-AAGCGTTCGTCCTGCACAA-3′; ECF2, 5′-TCATCCTTCGCCTCCCTG-3) for the species *P. aeruginosa* [30,31], with a positive control *P. aeruginosa* ATCC 27853^®^ and ATCC 9027^®^.

All PCR assays were performed in a 25 µL reaction mixture, containing 5x buffer (Promega, Madison, WI, USA), 25 mM MgCl_2_ (Promega, USA), 10 µM dNTPs (Bioline, Camarillo, CA, USA), 10 µM primers, 5 U/µL Taq DNA polymerase (Promega, USA) and sterile water. The reaction mixture was incubated in a thermocycler Veriti^TM^ Thermal Cycler (Applied Biosystems^TM^, Waltham, MA, USA). PCR amplifications started with an initial denaturation at 95 °C for 15 min, followed by 30 cycles of 95 °C for 45 s, 52 °C for 45 s, 72 °C for 45 s and then a final cycle at 72 °C for 7 min. PCR products were evaluated in 2.0% agarose gels with TBE 0.5X at 1.5% (*w*/*v*), with SYBR Gold (Invitrogen, Paisley, UK) and molecular marker (100 pb Promega, USA) at 100 V for 45 min.

(b) MALDI-TOF identification: Isolated colonies were identified using the Vitek MS Plus mass spectrometer (bioMerieux, Marcy l’Etoile, France) with the mass spectrum ranged from 2000 to 20,000 Da. A fresh colony isolated on trypticase soy agar (TSA) plates (Becton Dickson & Co., Cuautitlán Izcalli, Mexico) and incubated 24 h at 37 °C was directly spotted on the MALDI plate with the help of a sterile toothpick and placed onto a steel micro scout plate following the manufacturer’s instructions. After the plate was placed in the instrument and the system was operated using the method for the identification of bacteria. For each bacterial sample, mass fingerprints were processed by Compute Engine and the advanced spectrum classifier (ASC) algorithm of the Vitek MS system which automatically identifies a species by comparing the obtained spectrum (presence or absence of specific peaks) with the spectra typical of each claimed species (Vitek MS IVD version 3.0.0). A confidence interval of 98–99% was considered acceptable for species level identification (ID).

### 2.3. Detection of Virulence Factors

The same gDNA extracted from the cell lysis method was used for the virulence factor genes detection. All isolates were analyzed by PCR to identify the presence of eight virulence-related genes (*alg*D, *exo*S, *plc*H, *plc*N, *tox*A, *apr*A, *las*B and *rhl*AB) (Table 1) [32,33,34]. The PCRs were performed using 5× buffer (Promega, USA), 25 mM MgCl_2_ (Promega, USA), 10 µM dNTPs (Bioline, USA), 10 µM primers, 5 U/µL Taq DNA polymerase (Promega, USA) and sterile water in a total reaction volume of 25 µL. Four PCR reactions (three *multiplex* and one *singleplex*) were performed for each strain. The PCR program was 95 °C for 3 min, 30 cycles of 95 °C for 1 min, 55 °C for 45 s, 72 °C for 1 min 30 s and 72 °C for 5 min. PCR products were visualized in 2.0% agarose gels.

### 2.4. Antimicrobial Susceptibility Testing

The Kirby-Bauer disc diffusion method was employed according to the standard procedure described by the Clinical and Laboratory Standards Institute (CLSI) [35]. The isolates were tested against a panel of 16 antibiotics: piperacillin (P, 100 µg), piperacillin-tazobactam (TZP, 10/100 µg), ticarcillin (TIC, 10 µg), ticarcillin-clavulanic (75/10 µg, TIM), ceftazidime (30 µg, CAZ), cefepime (30 µg, FEP), aztreonam (30 µg, ATM), imipenem (10 µg, IPM), tobramycin (TM, 10 µg), meropenem (10 µg, MEM), gentamicin (10 µg, GM), amikacin (30 µg, AN), netilmicin (30 µg, NET), ciprofloxacin (5 µg, CIP), levofloxacin (5 µg, LEV) and norfloxacin (N, 10 µg). 

Isolates were inoculated onto trypticase soy agar (TSA) plates (Becton Dickson & Co., Cuautitlán Izcalli, Mexico) and incubated for 24 h at 37 °C. Bacterial suspensions were prepared from fresh culture into 0.9 saline water and turbidity was adjusted equivalent to a concentration of 0.5 McFarland. Each one was individually inoculated on Muller–Hinton agar plate (Becton Dickinson & Co., Franklin Lakes, NJ, USA) using a sterile cotton swab and antibiotic discs were dispensed on the surfaces of the inoculated plates, to then incubate at 37 °C for 24 h. After the incubation time, the diameter of the clear zone of inhibition around each antimicrobial disc was measured in millimeters (mm) and classified as resistant (R), intermediate (I) or susceptible (S) according to CLSI M100 document guidelines.

### 2.5. Detection Class 1 Integrons

All *P. aeruginosa* strains were analyzed by PCR to detect the presence of *intl*1 (encoding class 1) F: 5′-GGTCAAGGATCTGGATTTCG-3′/R: 5′-ACATGCGTGTAAATCATCGTC-3′ [36]. Negative controls (samples without a DNA template) and positive controls (samples with DNA from the collection of the Instituto Politécnico Nacional) were included in all PCR assays. The PCR reaction mixture was employed in 25 µL reaction volumes containing 5x buffer (Promega, USA), 25 mM MgCl_2_ (Promega, USA), 10 µM dNTPs (Bioline, USA), 10 µM primers, 5 U/µL Taq DNA polymerase (Promega, USA), DNA template and sterile water. PCR amplification was conducted following these conditions: 95 °C for 1 min, 30 cycles of 95 °C for 45 s, 54 °C for 45 s, 72 °C for 45 s and 72 °C for 7 min. PCR products were visualized in 2.0% agarose gels at 100 V for 45 min. A molecular marker was run concurrently (100 pb Promega, USA).

### 2.6. Detection of Heavy Metal Resistance Genes

Six heavy metal resistance genes were screened by PCR: genes encoding copper (*cop*A and *cop*B), arsenic (*ars*A and *ars*B), mercury (*mer*A) and cobalt/zinc/cadmium (*czc*A) (Table 2). The PCR reaction mixture contained 5x buffer (Promega, USA), 25 mM MgCl_2_ (Promega, USA), 10 µM dNTPs (Bioline, USA), 10 µM primers, 5 U/µL Taq DNA polymerase (Promega, USA) and DNA template. The volume of this mix was adjusted to 25 μL with sterile water. The PCR amplification conditions were as follows: initial denaturation at 95 °C for 1 min, followed by 30 cycles of denaturation at 95 °C for 45 s, annealing at 54–60 °C for 45 s, extension at 72 °C for 45 s and a final cycle of amplification at 72 °C for 7 min. Verification of PCR products was performed in horizontal electrophoresis using 2.0% agarose gel with 0.5× TBE and Sybr gold at 100 V for 45 min. A molecular marker was run concurrently (100 pb Promega, USA). The negative control consisted of all contents of the reaction mixture excluding template DNA, which was substituted with 1 μL sterile water. The DNA bands were visualized and photographed under UV light.

## 3. Results

### 3.1. Samples Collection

A total of 100 agricultural soil samples were collected from different locations in 24 municipalities from the state of Tamaulipas, Mexico. According to the USDA system, the texture of the samples was classified into 10 types of soil, mainly sandy clay loam (20%), clay loam (19%), silty loam (18%) and loam (16%). Soil samples were obtained from 12 different types of crops; the majority were collected from sorghum (29%), corn (24%), orange (16%) and sugar cane (13%).

### 3.2. Isolation and Identification of Pseudomonas aeruginosa

In the identification by molecular methods (PCR) of the 100 agricultural soil samples, the genus *Pseudomonas* was identified in 89% of samples and the *P. aeruginosa* species in 55%.

From each sample, between 1 and 3 strains were identified (depending on availability), making a total of 285 strains analyzed in this study. After molecular identification by MALDI-TOF, 125 strains of *Pseudomonas aeruginosa* were confirmed.

The 125 strains confirmed as *P. aeruginosa* were isolated from 55 soil samples, of which 27.2% (15/55) were silty loam, 23.6% (13/55) sandy clay loam and 16.3% (9/55) clay loam. Considering the type of crop present, 25.4% (14/55) were orange, 23.6% (13/55) corn, 21.8% (12/55) sorghum, 9.0% (5/55) sugar cane and six types of crops were in lower percentages (≤6.0%).

### 3.3. Detection of Virulence Factors

**Figure 1:**
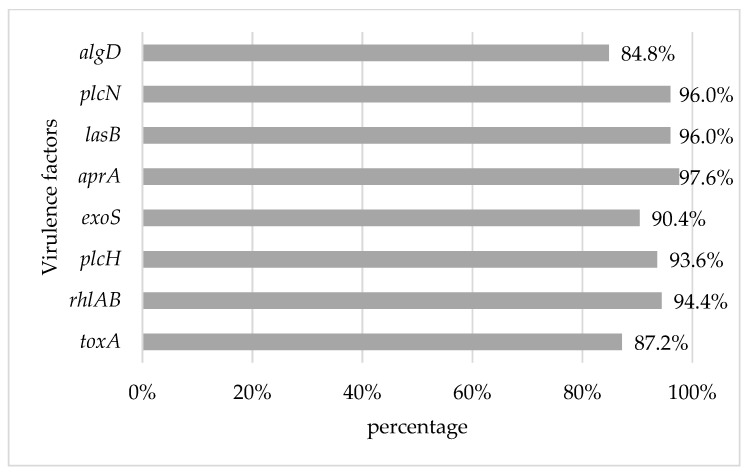
Prevalence of virulence factors in *P. aeruginosa* isolated from agricultural soil in Tamaulipas, Mexico.

The individual prevalence of the virulence factors analyzed was over 80% for the *P. aeruginosa* strains. The most prevalent virulence factor was *apr*A, present in 97.6% (122/125), followed by *las*B and *plc*N, both present in 96% (120/125) of the strains. On the other hand, the virulence factor with the lowest presence was *alg*D, present in 84.8% (106/125) of the strains (Figure 1). A total of 72% (90/125) of *P. aeruginosa* strains had the eight virulence factors analyzed.

### 3.4. Antimicrobial Susceptibility Testing

Among the 125 *P. aeruginosa* strains analyzed, 58.4% (73/125) showed resistance or intermediate resistance to at least one of the antibiotics tested. The strains were resistant or intermediate resistant to only 3 of the 16 antibiotics tested: ticarcillin, ticarcillin-clavulanic acid and aztreonam. A total of 32.8% (41/125) of the strains were intermediate resistant to ticarcillin, 4.0% (5/125) were resistant to ticarcillin/clavulanic acid, 36.8% (46/125) were intermediate resistant to ticarcillin/clavulanic acid, 0.8% (1/125) was resistant to aztreonam and 1.6% (2/125) were intermediate resistant to aztreonam. Considering both resistance and intermediate resistance, 2.4% (3/125) were co-resistant to TIM + ATM. While 14.4% (18/125) showed simultaneous intermediate resistance to TIM + TIC, no multiresistant strain was detected.

**Figure 2:**
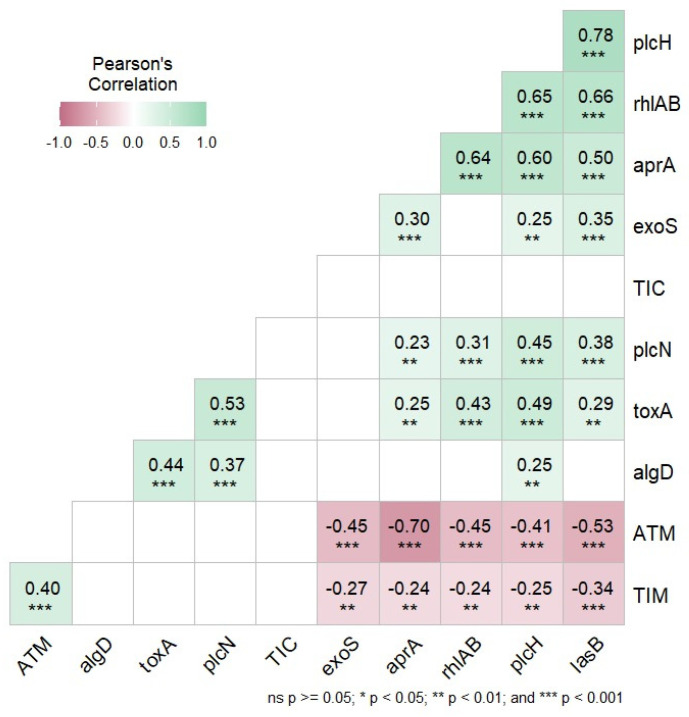
Correlation matrix between the antibiotics ticarcillin (TIC), ticarcillin-clavulanic (TIM), aztreonam (ATM), and the virulence factors. The intensity of the color is proportional to the correlation coefficient (−0.1 to 1.0), the * indicates significant Pearson correlation (*p* < 0.05), ** indicates moderate positive correlation and *** indicates strong positive correlation.

The results obtained between the antibiotics that showed some level of resistance (TIC, ATM and TIM) and the virulence factors were correlated (Figure 2). A strong positive correlation was observed between the ATM and *apr*A gene (−0.70 ***), ATM and *las*B (−0.53 ***), ATM and *exo*S (−0.45 ***), ATM and *rhl*A (−0.45 ***), ATM and *plc*H (−0.41 ***) and TIM and *las*B gene (−0.34 ***). In addition, a moderate positive correlation was detected between the TIM and *exo*S gene (−0.27 **), TIM and *plc*H (−0.25 **), TIM and *apr*A (−0.24 **) and TIM and *rhl*A gene (−0.24 **).

### 3.5. Detection Class 1 Integrons

The class 1 integrons (*int*1) were not identified in any of the 125 *P. aeruginosa* strains analyzed.

### 3.6. Detection of Heavy Metal Resistance Genes

**Figure 3:**
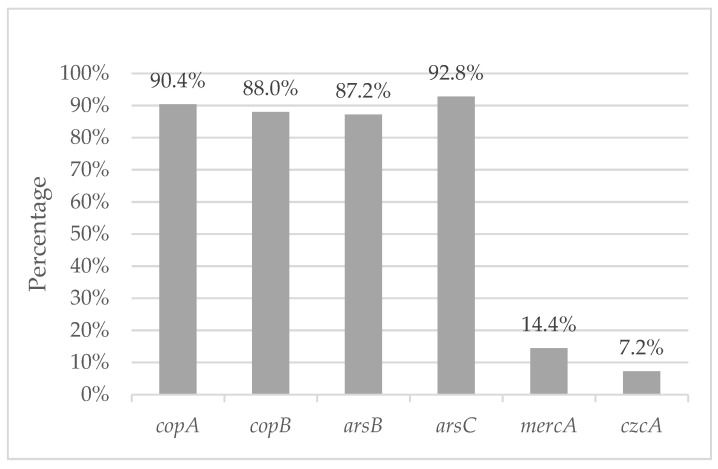
Prevalence of heavy metal resistance genes in *P. aeruginosa* isolated from agricultural soils.

The most prevalent heavy metal resistance genes were *ars*C and *cop*A, present in 92.8% (116/125) and 90.4% (113/125) of *P. aeruginosa* strains, respectively. However, *cop*B and *ars*B genes were also identified in a percentage higher than 80%, and the least prevalent genes were *mer*A, identified in 14.4% of the strains, and *czc*A, identified in 7.2% of the strains (Figure 3).

Considering the 73 strains that showed co-resistance to heavy metals and antibiotics (in the case of antibiotic resistance and intermediate resistance), 95.8% (70/73) exhibited one of the genes associated with resistance to copper (*cop*A or *cop*B). Of these, 90.4% (66/73) had the *cop*A gene and 90.4% (66/73) had *cop*B, while 84.9% showed both genes (*cop*A and *cop*B). Among the genes associated with arsenic resistance, of these strains 89.0% (65/73) presented the *ars*B gene and 93.1% (68/73) the *ars*C gene. In 84.9% (62/73), both genes (*ars*B and *ars*C) were detected, while the gene associated with resistance to mercury (*mer*A) was only detected in 12.3% (9/73) and the gene associated with resistance to cobalt/zinc/cadmium (*czc*) in 5.4% (4/73).

Now, considering the resistance to each antibiotic individually, among the 41 strains that showed intermediate resistance to TIC, 78.0% (32/41) had the *cop*A gene, 85.3% (35/41) the *cop*B gene, 80.4% (33/41) the *ars*B gene, 85.3% (35/41) the *ars*C gene, 12.1% (5/41) the *mer*A gene and 7.3% (3/41) the *czc* gene. Of the 51 strains that showed resistance or intermediate resistance to TIM, 88.2% (45/51) presented the *cop*A gene, 90.1% (46/51) *cop*B, 86.2% (44/51) *ars*B, 92.1% (47/51) *ars*C, 9.8% (5/51) *merc*A and 3.9% *czc*. Of the three strains that showed resistance or intermediate resistance to ATM and TIM, only one strain presented the *cop*A and *ars*C genes. In the other two strains, no genes associated with resistance to heavy metals were detected.

**Figure 4:**
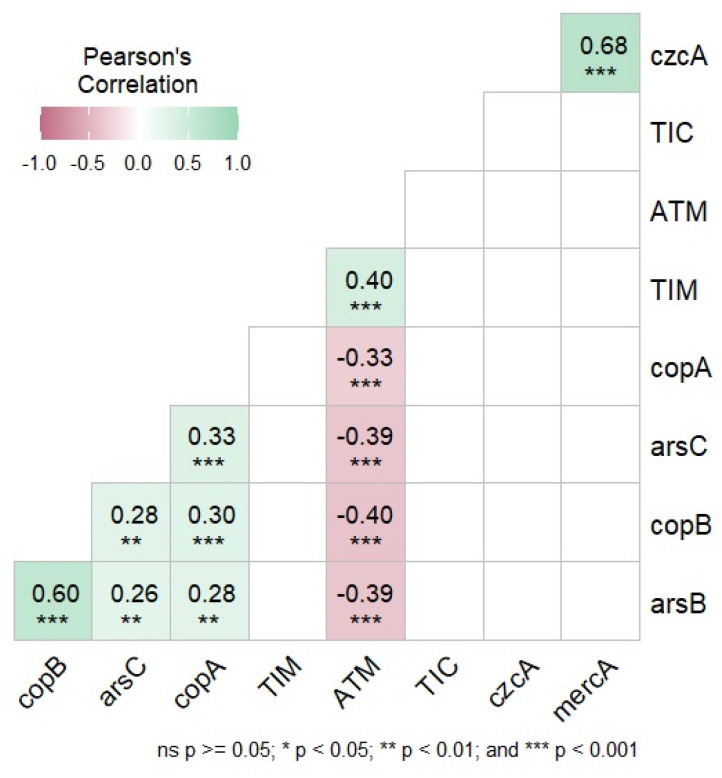
Correlation matrix between the antibiotics ticarcillin (TIC), ticarcillin-clavulanic (TIM), aztreonam (ATM) and the heavy metal genes. Values (Pearson’s R) are different from ‘zero’ with a significance level of *p* < 0.05). The * indicates significant positive correlation, ** indicates moderate positive correlation and *** indicates strong positive correlation.

The correlation between antibiotic resistance (TIC, ATM and TIM) and heavy metal genes (*ars*B, *ars*C, *cop*A, *cop*B, *czc*A, *merc*A) is represented in Figure 4. The results showed a strong positive correlation between the antibiotic aztreonam and the genes for copper (*cop*A with −0.33 *** and *cop*B with −0.40 ***) and arsenic (*ars*B with −0.39 *** and *ars*C with −0.39 ***). 

## 4. Discussion

*Pseudomonas aeruginosa* is a bacterium that can be found in various habitats due to its broad metabolic versatility that improves its distribution, proliferation and survival despite adverse physical and chemical conditions, thus enhancing its ecological success and potential threat to public health [39,40,41]. In this study, 100 agricultural soil samples were analyzed, and in 89% of the samples *Pseudomonas* spp. was detected, and 55% of those strains were confirmed as *P. aeruginosa*.

The prevalence or distribution of *Pseudomonas aeruginosa* may be due to several factors. Some authors have argued that bacteria are not randomly distributed, but are in different microenvironments (i.e., mainly in pores of different sizes and shapes) in the soil [42]. So, we consider the type of soil in which the strains were detected. Although 10 different types of soil were identified in the samples taken for this study, *P. aeruginosa* strains were isolated mainly from silt loam (15/18), sandy clay loam (13/20) and clay loam (9/19) soils. Another factor in the environment that could be related to the presence or absence of *P. aeruginosa* in the soil is the type of existing crop. Most strains were isolated from soil with orange (14/16), maize (13/24) and sorghum (12/29) crops. Statistically, the number of samples analyzed in this study is not sufficient to establish an association between the type of soil and the presence of *P. aeruginosa*, but these are the first results generated in the region. We hope that they will be a basis for directing studies in this objective.

In the current study for Tamaulipas, a prevalence of 55% of *P. aeruginosa* was detected, with results similar to those reported in other regions, such as 67% in Bangladesh [30] and 51.1% in Lithuania [43], but higher than what was published for the United States, with 37.5% [16], or India, with 33.8% [44].

*P. aeruginosa* is often described as a common soil bacterium [45]; however, it must be considered that its presence in agricultural soil can also be enriched by irrigation water, wastewater or livestock feces. Several studies have detected the presence of *P. aeruginosa* in surface waters and wastewater in a concentration from 19% to 70% [42,46,47,48]. This high prevalence indicates sites closely associated with human activities [45].

Along the course of a river or irrigation canal, water may mix with drainage water from domestic, industrial or other sources, increasing the possibility of transporting and accumulating bacteria such as *P. aeruginosa* in agricultural soil.

In Mexico, a previous study by Gutiérrez and collaborators [49] analyzed irrigation water samples in Michoacan and reported a 46% prevalence of *P. aeruginosa*. This is a similar percentage to what we detected in the current soil study for Tamaulipas (55%). Although in our study we did not include an analysis of the prevalence of *P. aeruginosa* in irrigation water, it is clear that irrigation water is an important factor influencing its presence in soil. Therefore, we will carry out a continuity study with rivers and irrigation canals in Tamaulipas.

Although a prevalence of *P. aeruginosa* greater than 50% was detected in agricultural soil samples from Tamaulipas, this does not necessarily imply a risk to public health. The pathogenicity of these strains depends on the virulence factors (VF) present, which allow bacterial colonization, penetration, damage to host tissues and successful infection [50]. Thus, only by knowing the factor virulence present can the potential risk that these strains represent for public health be evaluated.

In this study, 100% (125/125) of the strains *P. aeruginosa* had at least 2 VFs, 97.6% (122/125) had more than 3 VFs and 72.0% (90/125) had the totality of the VFs (8) analyzed. This result is higher compared to the strains analyzed in Brazilian agricultural soils, where 46% showed 1 to 3 VFs, and 39% presented 4 to 6 VFs [33]. In the current results for Tamaulipas agricultural soil, all analyzed VFs had a prevalence greater than 80%.

Toxin A (*tox*A), which can suppress protein synthesis and affect macrophage action, was present in 87.2% of the soil isolates in this study. This prevalence is lower than that reported by Gutierrez et al. [49] for irrigation water in Michoacan, Mexico, where they report the *tox*A gene in 100% of the strains analyzed. By contrast, alkaline protease (*apr*A), which can interfere with fibrin formation and inactivate host defense proteins, was detected in 97.6% of agricultural soil strains. This is similar to the 95.3% reported by Kaszab et al. [51] in environmental samples from Germany (soil, groundwater, compost, wastewater). For its part, elastase B exoenzyme (*las*B), an elastolytic zinc metalloproteinase and one of the virulence factors necessary for the process of initial pathogenesis and elastin degradation, was present in 96.0% of the strains. This percentage is like that reported by Kaszab et al. [51] (100%) or Gutierrez et al. [49] (98.0%) in environmental samples.

These virulence factors have been reported in high percentages in isolates from clinical samples, being associated with infections in humans, so we can assume that their presence in environmental isolates may represent a risk to public health. For example, Ghanem et al. [52] reported from different clinical specimens in Egypt the identification of *toxA* in 76.8%, *apr*A in 85.6%, *las*B in 89.6%, *alg*D in 80% and *exo*S in 84%. For their part, Vlada et al. [53] reported 100% of *apr*A and 96.7% of *las*B for clinical samples from India. Although the presence of these virulence factors gives the strain the ability to develop an infection, its concomitant susceptibility to antibiotics will make it possible to determine whether such an infection can be easily treated or complicated to the point of death. 

Resistant profiles of *P. aeruginosa* showed resistance or intermediate resistance to only 3 antibiotics of the 16 tested: ticarcillin (TIC, 32.8%), ticarcillin/clavulanic acid (TIM, 40.8%) and aztreonam (ATM, 2.4%), showing susceptibility to the rest of the antibiotics. 

While strains with some level of resistance to the antibiotics ticarcillin and ticarcillin clavulanic acid were isolated from samples from all over the state of Tamaulipas, the strains that were resistant to aztreonam were only obtained from the south of the state (municipalities of Mante and Altamira with silt loam soil growing sorghum and onion), without being found in any other area.

A study for agricultural soil in Brazil tested a panel of antibiotics similar to the current study in Tamaulipas, Mexico [54]. Coincidentally, they also report *P. aeruginosa* strains resistant to ATM and TIM, but in a much higher percentage (ATM, 92.5% and TIM, 85%). Furthermore, the strains from Brazil showed a low percentage of resistance (≤10%) to MEM, TET and PBM, which the strains from Tamaulipas did not present.

Previous studies in other geographic areas showed differences with respect to resistance profile. For example, *Pseudomonas* spp. isolated from agricultural fields in Lithuania showed no resistance against TIM, but high resistance to ATM (86%), and displayed low resistance against other antibiotic families like carbapenemics [43]. In contrast, *P. aeruginosa* strains isolated from soil associated with an industrial area in Bangladesh showed 0% of resistance to ATM, CAZ and CIP, but greater resistance (40–100%) to GM, PC, MEM, AN and IPM. 

Now, if the *P. aeruginosa* strains isolated from agricultural soil in Tamaulipas showed resistance to ATM and TIC, Gutierrez et al., [49] by contrast, did not report any resistance to these antibiotics in strains from irrigation water in Michoacan. However, while the strains in the current study only showed some level of resistance to 3 antibiotics, resistance to 11 antibiotics was observed in strains from irrigation water from Michoacan.

However, although the Tamaulipas strains only showed some level of resistance to 3 antibiotics, ATM and TIC are commonly used in clinical practice, especially in the treatment of patients with cystic fibrosis [54].

On the other hand, among the *P. aeruginosa* strains in the current study, no multi-antibiotic resistance (MDR) was detected. This agrees with the absence of the class 1 integron in the strains analyzed, since it has been established that integrons act as the main reason for multiple resistance in Gram-negative bacteria [55,56]. The absence of MDR strains and the high levels of susceptibility to most of the antibiotics tested may indicate that an infection caused by these strains could have effective treatments. This is a positive result, considering that the treatment of *P. aeruginosa* is often challenging because the organism has an intrinsic resistance to many antibiotics and can acquire resistance during therapy through various mechanisms [57]. 

As part of the results, a Pearson correlation analysis was performed between the antibiotics that showed some level of resistance (TIC, TIM and ATM) and the virulence factors. Only strong correlation (***) and moderate correlation (**) values were observed between the antibiotics ticarcillin-clavulanic acid (TIM), aztreonam (ATM) and some of the virulence factors. For ticarcillin (TIC), there was no significance value in relation to any of the virulence factors analyzed. Among the virulence factors, only *exo*S, *plc*H, *apr*A, *las*B and *rhl*AB presented strong or moderate correlation values with TIM or ATM, while *alg*D, *plc*N and *tox*A did not show significant values with any antibiotic.

The prevalence of antimicrobial-resistant strains depends on several factors. In recent years, the development of antibiotic resistance has been related with resistance to heavy metals, since they are encoded by genes that are physically linked to mobile genetic elements [58]. More importantly, heavy metals can induce selective pressure on microbial populations, leading to antimicrobial resistance through a mechanism called “co-selection” [59]. 

The *P. aeruginosa* strains isolated in the current study showed the presence of genes associated with heavy metal resistance, mainly to arsenic (*ars*B and *ars*C: 80–92.8%) and copper (*cop*A and *cop*B: 80–90%). As far as we reviewed, we did not find any studies in Mexico with which to compare the current results. However, the study carried out by Pitondo et al. [33] in Brazil also coincides with ours, identifying the highest frequency of genes associated with arsenic and copper. On the other hand, the gene *czc*A is related to resistance to cadmium, zinc and cobalt, which in Brazil registered a presence of 46%, and in the current study of Tamaulipas were only detected in 7.2% of the strains. For the gene *merc*A associated with resistance to mercury, in both studies it showed low prevalence (Brazil 2.3% and in Tamaulipas 14.4%).

A Pearson correlation analysis was performed between antibiotic resistance (TIC, ATM and TIM) and heavy metal genes (*ars*B, *ars*C, *cop*A, *cop*B, *czc*A, *merc*A). The results showed correlation values only between the antibiotic aztreonam and the genes associated with copper and arsenic. The antibiotics ticarcillin (TIC) and ticarcillin-clavulanic (TIM) did not show any positive correlation values with any of the heavy metal resistance genes. While the genes associated with copper and arsenic resistance showed positive correlation values, the genes *czc*A and *merc*A did not show any positive correlation values.

To our knowledge, these are the first published data for antibiotic and heavy metal resistance in *Pseudomonas aeruginosa* of environmental origin in Tamaulipas. To better understand the results obtained, monitoring will be carried out including the characteristics of the agricultural soil, the type of crop and the antibiotic concentration.

## 5. Conclusions

The results show that agricultural soil in Tamaulipas is a reservoir of *Pseudomonas aeruginosa* strains with a high prevalence of virulence factors, with the potential to cause infections. However, these strains analyzed showed high levels of resistance to only three antibiotics, indicating that potential infections could be effectively treated.

## Figures and Tables

**Table 1:** Primers of virulence-related genes used in the study.

Target Genes	Size (bp)	Primer Sequence 5′ → 3′	Reference
*alg*D	1310	ATGCGAATCAGCATCTTTGGT CTACCAGCAGATGCCCTCGGG	[32]
*exo*S	504	CTTGAAGGGACTCGACAAGG TTCAGGTCCGCGTAGTGAAT	[32]
*plc*H	307	GAAGCCATGGGCTACTTCAA AGAGTGACGAGGAGCGGTAG	[32]
*tox*A	352	GGTAACCAGCTCAGCCACAT TGATGTCCAGGTCATGCTTC	[32]
*apr*A	140	ACCCTGTCCTATTCGTTCC GATTGCAGCGACAACTTGG	[34]
*las*B	300	GGAATGAACGAAGCGTTCTC GGTCCAGTAGTAGCGGTTGG	[32]
*rhl*AB	151	TCATGGAATTGTCACAACCGC ATACGGCAAAATCATGGCAAC	[34]
*plc*N	466	GTTATCGCAACCAGCCCTAC AGGTCGAACACCTGGAACAC	[32]

**Table 2:** Primers of heavy metal resistance genes used in the study.

Target Genes	Size (bp)	Primer Sequence 5′ → 3′	Reference
*cop*A	475	CGGTCTCTACGAATACCGCTTCAA GAAATAGCTCATTGCCGAGGCGTT	[37]
*cop*B	364	TTCCTGCTCGACCAGTTGGAATAC GGTTGGTCAACAGGATGTCGTACT	[37]
*ars*B	410	GGTCTATGCGCTGGAGCAATTGAA TGCTGGGCATGTTGTTCATTACCG	[37]
*arsC*	205	GCAGCATTCTTTCCGAAGCCATGT TCGCAAACGGTGATGACGATGT	[37]
*mer*A	932	GTGCCGTCCAAGATCATGAT TAGCCYACRGTSGCSACYTG	[38]
*czc*A	206	GTTCACCTTGCTCTTCGCCATGTT ACAGGTTGCGGATGAAGGAGATCA	[37]

## Data Availability

The original contributions presented in the study are included in the article, further inquiries can be directed to the corresponding author.
